# Mitochondrial matrix chaperone and c-myc inhibition causes enhanced lethality in glioblastoma

**DOI:** 10.18632/oncotarget.16202

**Published:** 2017-03-15

**Authors:** Chiaki Tsuge Ishida, Chang Shu, Marc-Eric Halatsch, Mike-Andrew Westhoff, Dario C. Altieri, Georg Karpel-Massler, Markus David Siegelin

**Affiliations:** ^1^ Department of Pathology and Cell Biology, Columbia University Medical Center, New York, NY, USA; ^2^ Department of Neurosurgery, Ulm University Medical Center, Ulm, Germany; ^3^ Department of Pediatrics and Adolescent Medicine, Ulm University Medical Center, Ulm, Germany; ^4^ The Wistar Institute, Philadelphia, PA, USA

**Keywords:** gamitrinib, c-myc, OTX015, JQ1, apoptosis

## Abstract

Malignant gliomas display high levels of the transcription factor c-myc and organize a tumor specific chaperone network within mitochondria. Here, we show that c-myc along with mitochondrial chaperone inhibition displays massive tumor cell death. Inhibition of mitochondrial matrix chaperones and c-myc was established by utilizing genetic as well as pharmacological approaches. Bromodomain and extraterminal (BET) family protein inhibitors, JQ1 and OTX015, were used for c-myc inhibition. Gamitrinib was applied to interfere with mitochondrial matrix chaperones. A xenograft model was used to determine the *in vivo* efficacy. Combined inhibition of c-myc and mitochondrial matrix chaperones led to a synergistic reduction of cellular proliferation (CI values less than 1) in established glioblastoma, patient-derived xenograft and stem cell-like glioma cultures. The combinatorial treatment of BET inhibitors and Gamitrinib elicited massive apoptosis induction with dissipation of mitochondrial membrane potential and activation of caspases. Mechanistically, BET-inhibitors and Gamitrinib mediated a pronounced integrated stress response with a PERK-dependent up regulation of ATF4 and subsequent modulation of Bcl-2 family of proteins with down-regulation of Mcl-1 and its interacting partner, Usp9X, and an increase in pro-apoptotic Noxa. Blocking ATF4 by siRNA attenuated Gamitrinib/BET inhibitor mediated increase of Noxa. Knockdown of Noxa and Bak protected from the combinatorial treatment. Finally, the combination treatment of Gamitrinib and OTX015 led to a significantly stronger reduction of tumor growth as compared to single treatments in a xenograft model of human glioma without induction of toxicity. Thus, Gamitrinib in combination with BET-inhibitors should be considered for the development for clinical application.

## INTRODUCTION

Certain tumor types are highly treatment resistant and therefore require additional attention with regards to the design of novel treatments. Here, we propose a novel treatment strategy for the most common primary brain tumor, glioblastoma [1, 2].

Cancer cells display certain dependencies on metabolism. One of the most prominent observations is the reliance of tumor cells on “aerobic glycolysis”. This term was coined by the fact that tumor cells heavily depend on glycolysis despite the abundant presence of oxygen. Instead of metabolizing glucose via the citric acid cycle and subsequent oxidative phosphorylation, tumor cells convert glucose to lactate, producing only a much smaller amount of ATP. However, this inefficient energy production by tumor cells renders them susceptible to certain treatment strategies.

One oncogenic transcription factor that regulates tumor cell metabolism is c-myc. In malignant gliomas c-myc is commonly up regulated and therefore it is conceivable to target this transcription factor for therapy. Recently, c-myc became targetable through the introduction of BET-inhibitors, JQ1 and OTX015 [3, 4] that ultimately inhibit c-myc expression, causing inhibition of proliferation and selectively induction of apoptosis [3, 5–7]. Aside from c-myc, mitochondrial matrix chaperones are also known to control tumor cell metabolism [8] and the specific interference with these molecules by a small molecule, called Gamitrinib [9, 10], represents a viable therapeutic opportunity. Gamitrinib was first described in 2009 and is a molecule that consists of a Hsp90 antagonist linked to a mitochondrial targeting sequence to enable mitochondrial matrix chaperone inhibition [11]. Mitochondrial matrix chaperones, including Hsp90, were earlier found to be up-regulated in cancer cells and inhibition of these molecules leads to preferential tumor cell killing [12].

In this work, we demonstrate that inhibition of the mitochondrial matrix chaperones along with the oncogenic transcription factor, c-myc, is synthetically lethal in various model systems of glioblastoma, including tumor initiating cells.

## RESULTS

### Inhibition of mitochondrial matrix chaperones and c-myc causes synergistic reduction in cellular proliferation

**Figure 1:**
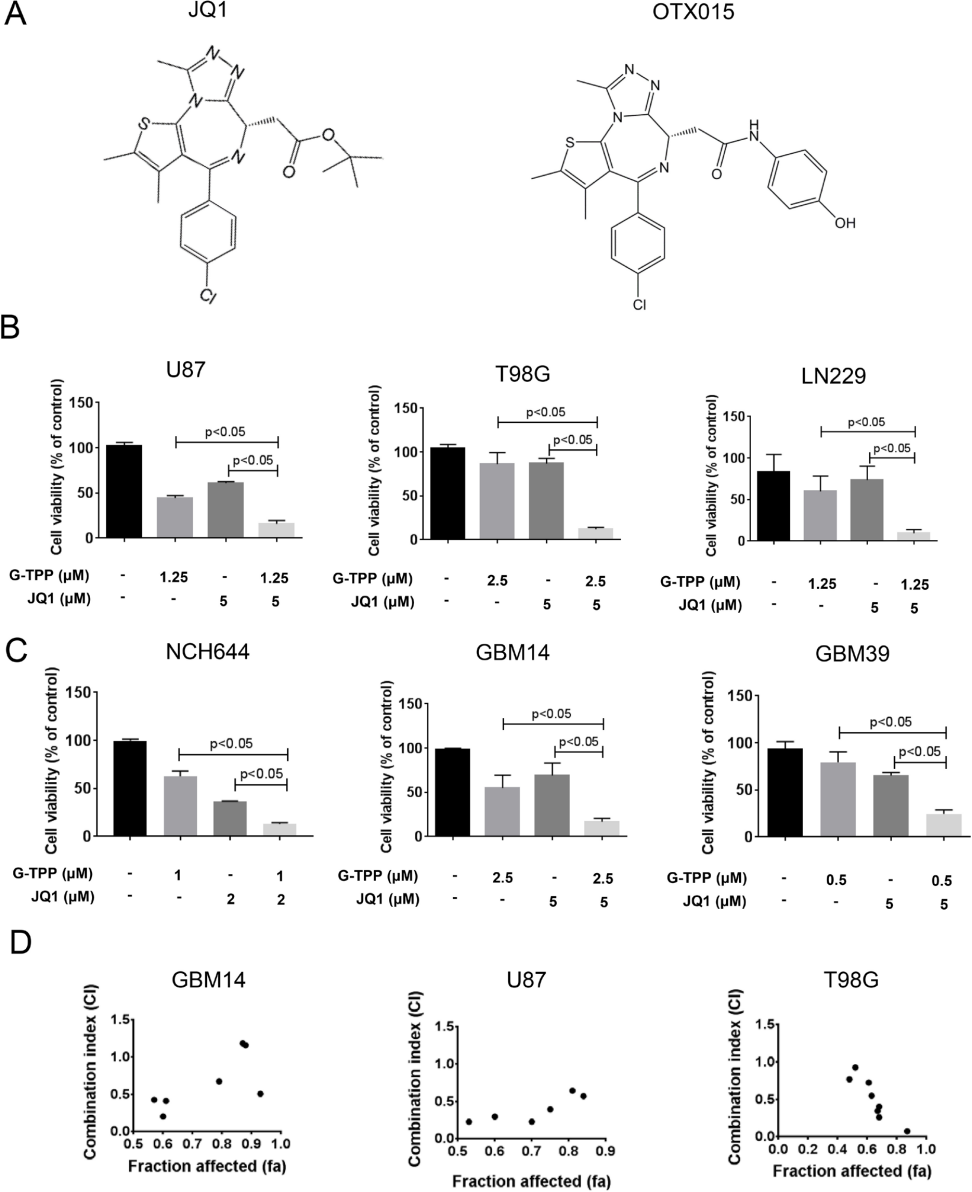
Combined treatment with Gamitrinib and the BET-inhibitor (JQ1 and OTX015) results in a synergistic antiproliferative effect across a wide spectrum of human glioma cells (**A**) Chemical Structure of BET-inhibitors, JQ1 and OTX015. (**B**–**C**) U87MG, T98G and LN229 established glioblastoma cell lines, NCH644 stem cell-like glioma and GBM14 and GBM39 patient-derived xenograft cultures were treated were treated with solvent, G-TPP, JQ1 or the combination of both. After 72 h of treatment, CellTiter-Glo assays were performed. Column: mean. Error bar: standard deviation (SD). *n* = 3. Statistical analysis was performed and *p* values were calculated. A *p*-value of less than 0.05 was considered statistically significant. (**D**) CI values and fraction affected were calculated in GBM14, U87 and T98G cells, using the CompuSyn software (ComboSyn, Inc., Paramus, NJ, U.S.A.). Data points located below 1 (CI value less than 1) indicate a synergistic drug-drug interaction and data points larger than 1 indicate an antagonistic drug-drug interaction. Some data points overlap and are therefore not represented on the graphical chart. For individual values, please refer to Table 1 or Supplementary Table 1.

We started this study in order to enhance the limited effects of BET-inhibitors. To this purpose, we hypothesized that interference with mitochondrial matrix chaperones and c-myc causes enhanced lethality in model systems of malignant glioma. We found that the simultaneous administration of JQ1 and the mitochondrial matrix chaperone inhibitor, Gamitrinib-TPP, causes enhanced reduction of proliferation in established glioblastoma cells (U87, T98G and LN229), stem cell-like glioma cultures (NCH644) and patient derived xenograft cultures (GBM14 and GBM39) and that this occurred in a statistically significant manner (Figure 1B and 1C). Comparable results were obtained when using a structural analogue, OTX015, in lieu of JQ1 (Supplementary Figure 1). Synergism analysis confirmed that over a broad range of concentrations JQ1 and G-TPP acted in a strong synergistic fashion in GBM14, U87 and T98G cells (Figure 1D, Table 1 and Supplementary Table 1).

**Table 1:** CI values for glioblastoma cultures after combinatorial treatments with G-TPP and JQ1

T98G		
G-TPP (μM)	JQ1 (μM)	CI
1.0	2.0	0.76819
2.0	3.0	0.92700
3.0	4.0	0.40197
4.0	5.0	0.07398
4.0	2.0	0.25957
3.0	3.0	0.34501
2.0	4.0	0.54819
1.0	5.0	0.72332

G-TPP treatment elicits a synergistic anti-proliferative effect on T98G cells in the presence of the c-myc inhibitor, JQ1. The CompuSyn software (ComboSyn, Inc., Paramus, NJ) was used for the drug-drug interaction analysis including the calculation of the combination index (CI). A CI < 1 was considered as synergistic, a CI = 1 as additive and a CI > 1 as antagonistic.

### The enhanced reduction of cellular viability by the combination treatment of G-TPP and BET-inhibitors is mediated by enhanced activation of apoptosis

**Figure 2:**
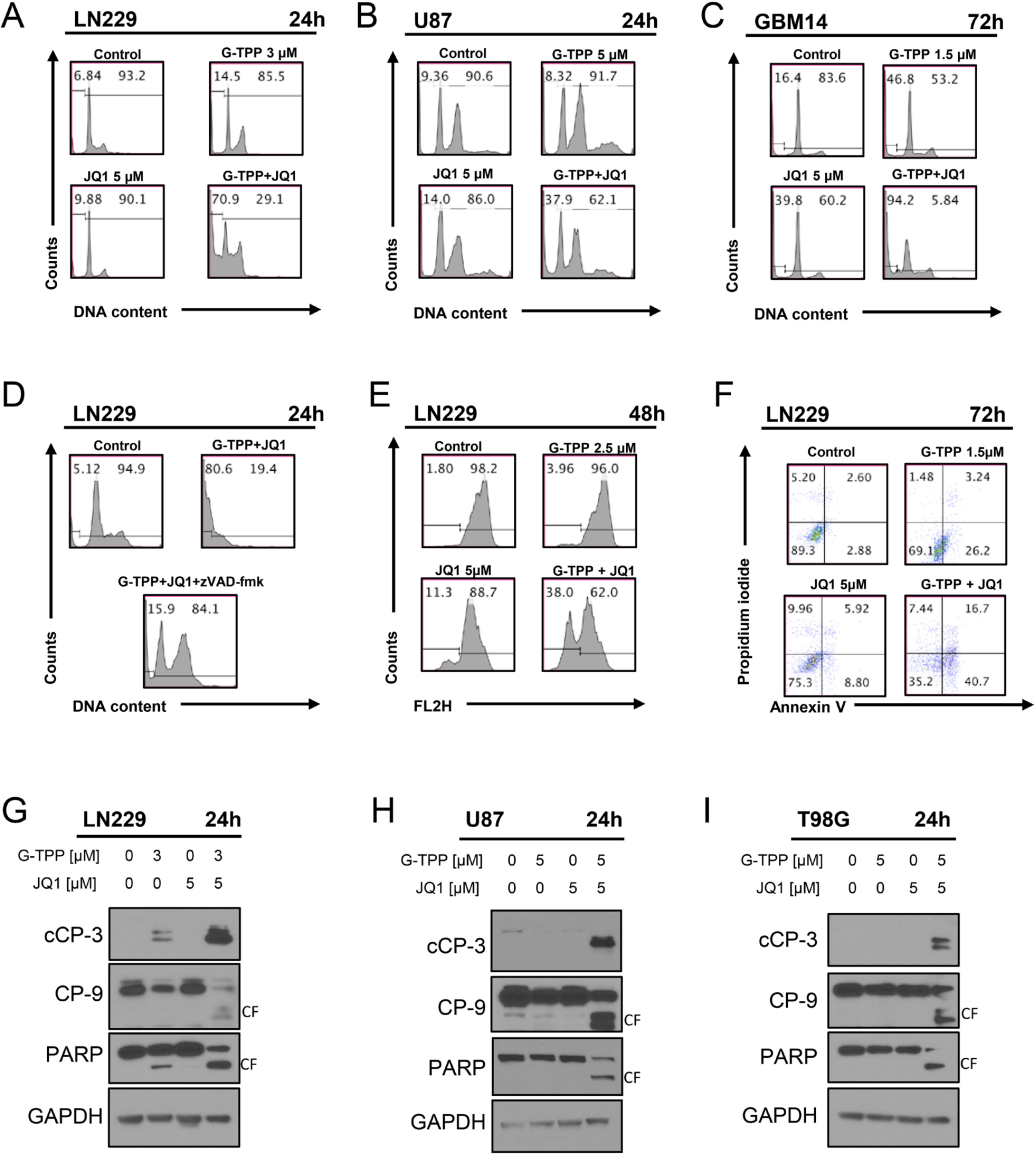
Combined treatment with JQ1 and G-TPP yields enhanced induction of apoptosis (**A**) (**B**, **C)** representative histograms of LN229, U87 and T98G glioblastoma cells that were treated for the indicated time points as indicated with JQ1, G-TPP or both prior to staining with propidium iodide and flow cytometric analysis. (**D**) LN229 cells were treated with solvent, G-TPP+JQ1 in the presence or absence of zVAD-fmk. Subsequently, samples were fixed and stained with Propidium iodide and analyzed by flow cytometry for DNA-fragmentation. Representative flow plots are shown. (**E**) LN229 glioblastoma cells were treated with G-TPP, JQ1 or the combination of both and subsequently stained with TMRE for the assessment of mitochondrial membrane potential. Flow cytometric analysis was performed and representative plots are shown. (**F**) LN229 glioblastoma cells were treated with solvent, G-TPP, JQ1 or the combination of both. Cells were stained with Annexin V/Propidium iodide and analyzed by flow cytometry. Representative plots are shown. (**G**–**I**) LN229, U87 and T98G cells were treated with solvent, G-TPP, JQ1 or the combination as indicated. Whole cell protein lysates were isolated and western blot analysis for the expression cleaved caspase-3 (cCP-3), caspase-9 (CP-9) and PARP was performed. GAPDH was used to confirm protein loading. CF: cleaved fragment.

Next, we determined the underlying mechanism as to how the drug combination of BET-inhibitors and Gamitrinib elicit their anti-proliferative effect. Based on the morphological appearance of the cells, we hypothesized that most likely apoptosis is involved. To this end, we analyzed the percentage of DNA – fragmentation by flow-cytometry in LN229, U87 and patient derived xenograft cells (GBM14) (Figure 2A–2C). To confirm apoptosis induction in another assay, we conducted Annexin V/PI staininig and found that the combination treatment led to enhanced staining with Annexin V as compared to control and single treatments (Figure 2F and Supplementary Figure 2). Consistently, LN229 glioma cells treated with the combination showed enhanced loss of mitochondrial membrane potential (Figure 2E). To confirm the involvement of caspases in the cell death induction by the combination treatment, LN229 cells were exposed to a caspase-inhibitor, zVAD-fmk and zVAD-fmk was partially protective for G-TPP/JQ1 mediated cell death (Figure 2D). In agreement with an involvement of caspases, we analyzed caspase cleavage products. The combination treatment led to an enhanced cleavage of PARP, caspase-3 and caspase-9 in LN229, U87 and T98G cells (Figure 2G–2I). All in all, these findings suggest that apoptosis is the main cell death mechanism elicited by the combination treatment.

### The combination treatment of Gamitrinib and BET-inhibitors causes a modulation of Bcl-2 family of proteins

**Figure 3:**
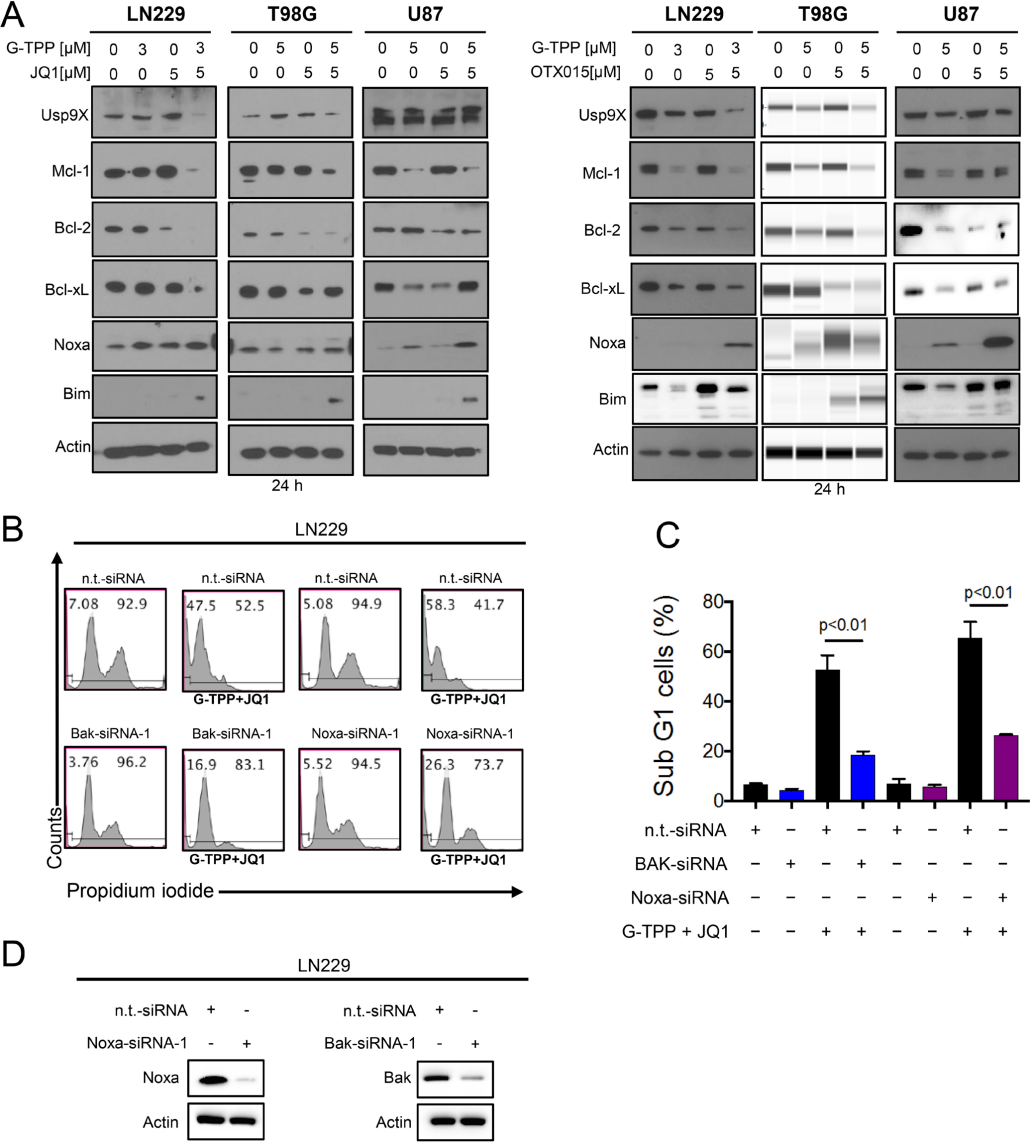
Treatment with G-TPP, BET-inhibitors and the combination treatment of BET-inhibitors and G-TPP modulates protein expression of the Bcl-2 family of proteins (**A**) LN229, T98G and U87 cells were treated with G-TPP, BET-inhibitors (JQ1 or OTX015) or the combination of both for 24 h. Whole cell extracts were collected and Western blot analysis was performed for Usp9X, Mcl-1, Bcl-2, Bcl-xL, Noxa and Bim. Actin served as loading control. T98G cells treated with G-TPP, OTX015 or the combination was analyzed on a capillary electrophoresis system (Wes). (**B**) LN229 cells were transfected with non-targeting, Noxa or Bak specific siRNAs and subsequently exposed to G-TPP+JQ1. Samples were then harvested, stained with Propidium iodide and DNA – fragmentation was analyzed by flow cytometry. Representative plots are shown. (**C**) Shown are the quantifications for the experiment in B. Column: mean. Error bar: standard deviation (SD). *p* < 0.01. (**D**) LN229 cells were transfected as described in B. Western Blot analysis was performed to confirm Bak and Noxa protein suppression. Actin serves as a loading control.

Due to the activation of the apoptotic pathway, we hypothesized that the expression of the Bcl-2 family of proteins might be altered by the various treatments. To this purpose, we assessed the expression of Bcl-2 family members in response to the various treatments, including two structurally analogous BET-inhibitors, in LN229, U87 and T98G. We found that Mcl-1 and Bcl-2 protein levels were suppressed after treatment with the combination of G-TPP+OTX015 and G-TPP+JQ1 in LN229, T98G and U87 cells (Figure 3A). In contrast, Bcl-xL was not as consistently regulated. The deubiquitinase Usp9X was down regulated in most instances except in U87 after treatment with G-TPP+JQ1. With respect to the pro-apoptotic Bcl-2 family members, we also determined the expression of Noxa after the various treatments. We found that Noxa was consistently up regulated by the combination treatment (G-TPP+OTX015) in LN229, T98G and U87 (Figure 3A). Similar results were obtained when using JQ1 in lieu of OTX015 (Figure 3A). Although in T98G cells Noxa was not elevated by the combination treatment of G-TPP and JQ1, Mcl-1 protein levels were down regulated, elevating the Noxa/Mcl-1 ratio, favoring apoptosis (Figure 3A). Aside from Noxa elevation, the combinatorial treatment up regulated Bim protein levels (Figure 3A), which is known to facilitate intrinsic apoptosis.

### Knockdown of Noxa and Bak protects from cell death induced by the combination treatment of Gamitrinib and BET-inhibitors

Given that Noxa was increased by the combination treatment we determined to which extend Noxa contributes to the combination treatment of BET-inhibitors and G-TPP. For this purpose, LN229 cells were transfected with Noxa specific siRNA and suppression of Noxa was confirmed by immunoblotting (Figure 3D). 72 h after transfection with either non-targeting or Noxa specific siRNA LN229 cells were treated with the drug combination of G-TPP and JQ1. LN229 cells transfected with Noxa specific siRNA demonstrated less cell death induction when compared to non-targeting siRNA transfected cells (Figure 3B and 3C). Given that Noxa antagonizes the function of Mcl-1 and Mcl-1 preferentially interacts with Bak, we tested the hypothesis that knockdown of Bak is protective from cell death induction by the combination treatment of G-TPP and JQ1. LN229 cells that were transfected with a Bak specific siRNA demonstrated reduced protein levels of Bak as compared to cells transfected with non-targeting siRNA (Figure 3D). 72 h after transfection with either Bak or non-targeting siRNA LN229 cells were treated with the drug combination therapy of G-TPP and JQ1. In agreement with our hypothesis, LN229 cells with silenced Bak levels were more resistant towards the combination treatment (Figure 3B and 3C).

### The combination treatment elicits an integrated stress response with evidence for endoplasmic reticulum stress

**Figure 4:**
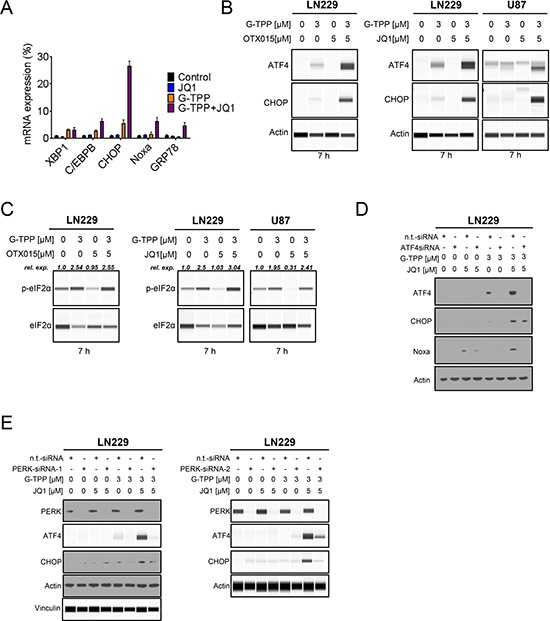
The combination treatment of BET-inhibitors and Gamitrinib elicits enhanced endoplasmic reticulum stress (**A**) LN229 cells were treated with solvent, JQ1, G-TPP or the combination of both for 7 h. Subsequently, RNA was isolated and real-time PCR analysis was performed for makers of ER-stress: XBP1, C/EBPB, CHOP, GRP78 and ATF4 downstream effector Noxa (PMAIP1). (**B**) LN229 cells were treated with G-TPP, OTX015 or the combination of both for 7 h. LN229 and U87 cells were treated with G-TPP, JQ1 or the combination of both for 7 hours. Whole cell protein lysates were isolated and analyzed by capillary electrophoresis (Wes). (**C**) The same protein lysates as described under B were analyzed by capillary electrophoresis (Wes) for the expression of p-eIF2α and total eIF2α. The quantifications were performed in Compass Software (Wes Instrument). (**D**) LN229 cells were transfected with non-targeting or ATF4 specific siRNAs. 72 h after transfection LN229 cells were treated with G-TPP, JQ1 or the combination of both for 7 hours. Subsequently, whole cell protein lysates were isolated and analyzed by western blotting for the expression of ATF4, CHOP and Noxa. Actin serves as loading control. (**E**) LN229 cells were transfected with non-targeting or two PERK specific siRNAs. 72 h after transfection LN229 cells were treated with G-TPP, JQ1 or the combination of both for 7 hours. Subsequently, whole cell protein lysates were isolated and analyzed by western blotting for the expression of ATF4 and CHOP. Data for PERK-siRNA-2 were analyzed by capillary electrophoresis (Wes). Data for PERK-siRNA-1 were analyzed by conventional western blotting except ATF4 and Vinculin, which were analyzed by capillary electrophoresis (Wes).

Based on our findings that the combination treatment increased the protein levels of Noxa and Bim, we hypothesized that this effect might be mediated through an integrated stress response, which most likely originated in the endoplasmic reticulum (ER). To this end, LN229 glioblastoma cells were treated with JQ1, G-TPP and the combination of G-TPP and JQ1. After 7 h, RNA was isolated and mRNA expression for markers of ER-stress was determined. The combination treatment elicited a significant increase in GRP78 (BIP), suggesting activation of ER-stress. In contrast, single treatments (JQ1 and G-TPP) elicited a smaller increase (Figure 4A). In keeping with this finding, other ER-stress mediators, such as XBP1, C/EBPB and CHOP were up regulated as well (Figure 4A). Transcript levels for Noxa were also increased by the combination treatment (Figure 4A).

### The combination treatment of Gamitrinib and BET-inhibitors leads to a PERK-dependent increase of ATF4

Given that ATF4 is a major downstream regulator of the ER-stress response [13], we assessed as to whether G-TPP, BET-inhibitors and the combination of both up regulate the protein levels of ATF4 and its downstream target CHOP. The combination treatment of JQ1 and G-TPP led to a pronounced increase in both ATF4 and CHOP levels in LN229 cells 7 h after treatment (Figure 4B). This finding was recapitulated when the structural relative, OTX015, was used in lieu of JQ1 (Figure 4B). We also confirmed that the enhanced increase of ATF4 and CHOP protein levels by the combined treatment of G-TPP and JQ1 was also seen in other cell lines (U87) (Figure 4B). Moreover, the ER-stress response was sustained (CHOP protein elevation) and was still detectable 24 hours after treatment (Supplementary Figure 3A and 3B). ATF4 expression is regulated by eif2α. When eif2α is phosphorylated at Serine 51 [14], certain mRNAs are preferentially translated, including ATF4. We found that the combination treatment of BET-inhibitors, JQ1 and OTX015, enhanced phosphorylation of eif2α in LN229 and U87 cells 7 h after treatment, which is in keeping with the up regulation of ATF4 under these conditions (Figure 4C). Next we assessed as to whether or not CHOP and Noxa, which are regulated by ATF4 and are known to mediate cell death, are increased by the combination treatment in an ATF4 dependent manner. To this purpose, we silenced the expression of ATF4 in LN229 and subsequently treated the cells with G-TPP, JQ1 or the combination of G-TPP and JQ1 for 7 hours. As previously observed in Figure 4B, we found that only G-TPP and the combination treatment of G-TPP and JQ1 increased ATF4 protein levels, which was abolished by the ATF4 siRNA (Figure 4D). Consistently, siRNA mediated suppression of ATF4 also attenuated the increase of Noxa mediated by the combination treatment, suggesting that Noxa levels are controlled by ATF4 (Figure 4D). Because eif2α was phosphorylated at Serine 51 upon treatment with G-TPP and the combination treatment of G-TPP and JQ1, we reasoned that the ATF4 increase mediated by the combination treatment is likely mediated in a PERK-dependent manner, since PERK is known to phosphorylate eif2α at Serine 51. To confirm this hypothesis, we transfected LN229 cells with two specific siRNAs, targeting PERK. Knockdown was confirmed by immunoblotting (Figure 4E). After transfection LN229 cells were treated with G-TPP, JQ1 or the combination of both for 7 h. We found that knockdown of PERK potently abrogated the increase of ATF4 mediated by the combination treatment of G-TPP and JQ1 (Figure 4E). In keeping with the regulation of ATF4, knockdown of PERK also suppressed CHOP up regulation (Figure 4E).

### Specific silencing of c-myc is sufficient to sensitize cells for Gamitrinib-mediated cell death

**Figure 5:**
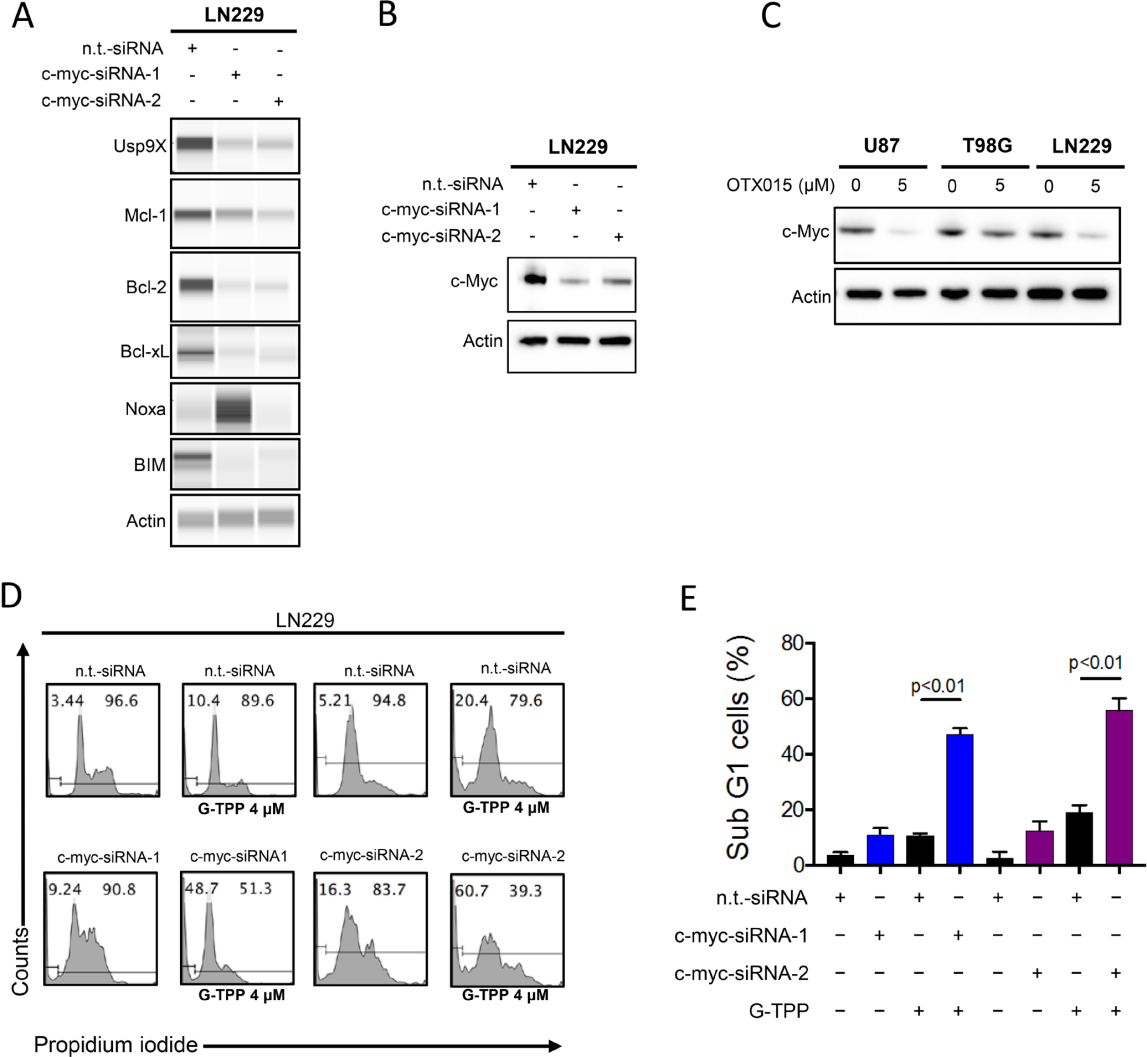
Silencing of c-myc sensitizes for Gamitrinib mediated cell death (**A**) LN229 cells were transfected with n.t.-siRNA, c-myc-siRNA-1 or c-myc-siRNA-2. Whole cell protein lysates were prepared. Protein expression for Usp9x, Mcl-1, Bcl-2, Bcl-xL, Noxa and Bim were analyzed by capillary electrophoresis. Actin serves as a loading control. (**B**) LN229 cells were transfected as in A. Protein expression for c-myc was determined by western blotting. Actin serves as a loading control. (**C**) U87, T98G and LN229 cells were treated with OTX015 for 72 h. Subsequently, whole protein lysates were prepared and protein expression for c-myc was determined by Western Blotting. Actin serves as a loading control. (**D**) LN229 cells were transfected as described in A. Subsequently, LN229 cells were treated with G-TPP. 24 hours after treatment, cells were fixed and stained with propidium iodide. DNA-fragmentation was analyzed by flow-cytometry. (**E**) Quantification of the results in D. Column: mean. Error bar: SD.

The major effect of OTX015 and JQ1 is the suppression of c-myc protein levels. In agreement with this fact we found that OTX015 suppresses c-myc protein levels in U87, LN229 and T98G cells, confirming that the key target is affected by the drug in our model systems (Figure 5C). Next, we hypothesized that JQ1 and OTX015 sensitize for Gamitrinib mediated cell death through their inhibitory effect on c-myc. To this end, we utilized two c-myc specific siRNAs. LN229 cells that were transfected with c-myc specific siRNA 1 and 2 revealed a potent reduction in c-myc protein levels (Figure 5B). In addition, c-myc knockdown also affected the expression level of the Bcl-2 family of proteins, Mcl-1, Bcl-2, Bcl-xL as well as the proapoptotic members, Noxa and BIM (Figure 5A). Moreover, suppression of c-myc levels is sufficient to sensitize LN229 cells to Gamitrinib mediated cell death (Figure 5D and 5E), suggesting that c-myc suppression is a main contributor of BET-inhibitor mediated sensitization to Gamitrinib mediated cell death.

### The combination treatment of Gamitrinib and BET-inhibitor, OTX015, displayed a regression of tumors *in vivo*

**Figure 6:**
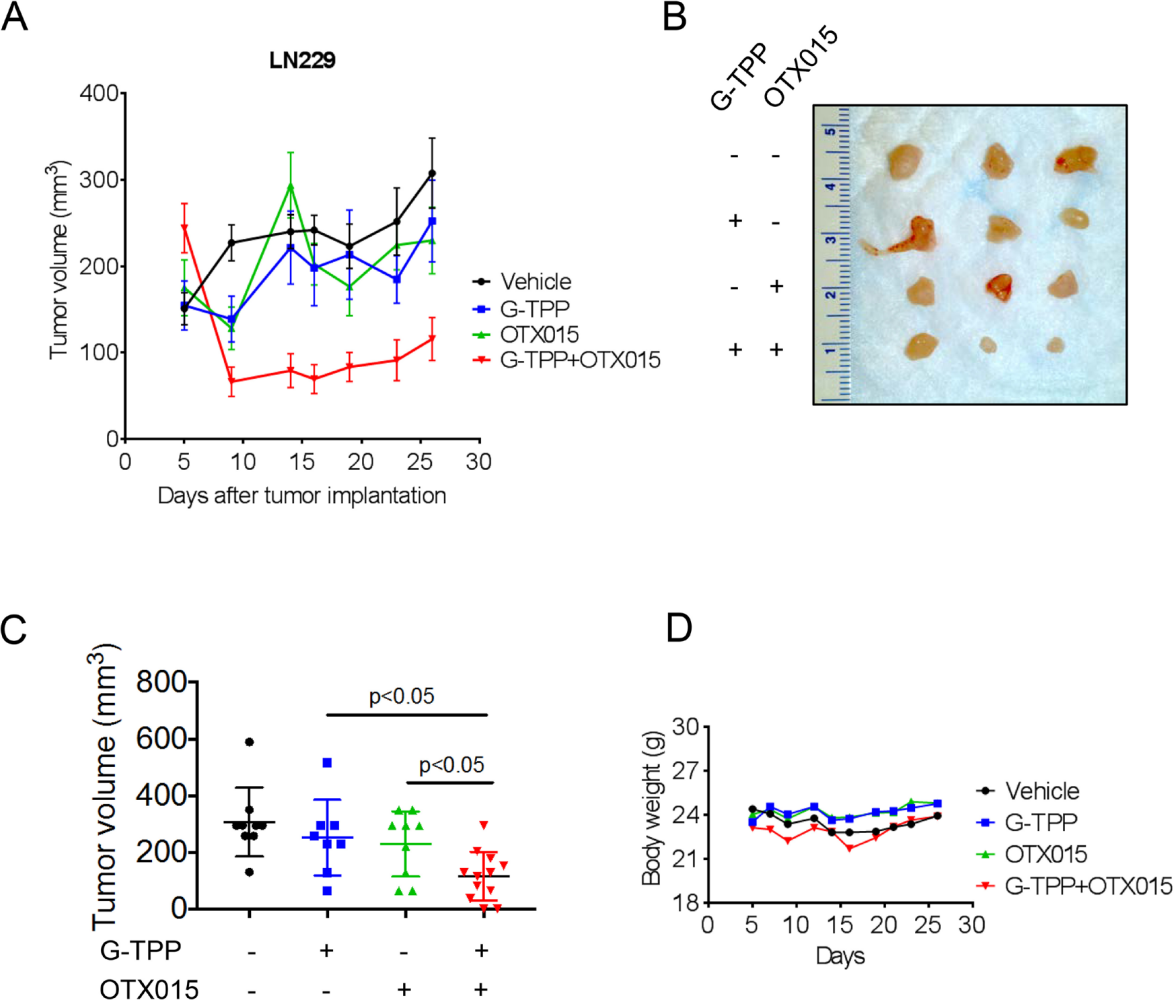
Combined treatment with G-TPP and OTX015 leads to a regression of glioblastoma xenograft tumors (**A**–**D**) 1 × 10^6^ LN229 glioblastoma cells were implanted subcutaneously. After tumor formation, groups were formed. Animals were treated intraperitoneally with vehicle (*n* = 9), OTX015 (50 mg/kg) (*n* = 9), G-TPP (5 mg/kg) (*n* = 9) or both agents (*n* = 12) (5 days on, 2 days off in week 1 and 2; 3 times a week in week 3 for 3 weeks. Tumor growth curves show the development of tumor size for each treatment group. Data are presented as mean and SEM. B, Photograph of representative tumors is shown. C, Scatter plots display the quantitative representation of the tumor size among the different treatments toward the end of the experiment. Data are presented as mean and SD. D, Body weights of the animals are provided throughout of the treatment (B). Data are presented as means.

To assess the efficacy of the combination treatment of OTX015 and Gamitrinib, we tested this drug combination in a heterotopic glioblastoma xenograft model. To this end, LN229 glioblastoma cells were implanted subcutaneously. After the appearance of tumors, four treatment groups were formed and treatment was initiated for 3 three weeks. While the single treatments, Gamitrinib and OTX015, and vehicle did not elicit a significant reduction in tumor growth, the combination treatment of Gamitrinib-TPP and OTX015 resulted in a statistically significant growth reduction with partial regression of tumors (Figure 6A and 6C). After finishing the experiments, tumors were resected and gross images were taken, showing that the combination treatment had smaller tumors than vehicle or single treatments (Figure 6B). With regards to potential toxicity, weight measurements were taken throughout the treatment and we did not detect any significant weight loss in the treatment groups, suggesting that the proposed drug combination is well-tolerated and displays little side effects.

## DISCUSSION

In this report, we discovered a novel synthetic lethal interaction between the inhibition of mitochondrial matrix chaperones and c-myc. Treatment with Gamitrinib and BET-inhibitors led to a significant synergistic reduction in cellular viability in a range of model systems of glioblastoma, including stem cell-like glioma cells. Stem cell-like glioma cells remain a centerpiece in glioblastoma research [15] because they are known to drive tumor recurrence and treatment resistance. Therefore, around a decade ago, it became evident that treatments that do not target the stem cell niche will likely fall short of expectations [16]. BET-inhibitors appear to be a welcome contribution for targeting the stem cell fraction in these neoplasms since it was shown earlier that brain tumor initiating cells express high levels of c-myc [17] and are therefore dependent on this transcription factor. However, due to the pronounced heterogeneity of glioblastomas, interfering with c-myc will likely not suffice to keep these tumors in check. Therefore, others and we investigate further strategies to identify novel combination treatments and it appears that dual inhibition of c-myc along with mitochondrial matrix chaperones is a viable approach to kill glioblastoma cells in a synergistic manner.

Concerning the cell death mechanisms, we found that the combination treatment of Gamitrinib and BET-inhibitors [3, 4] elicits enhanced apoptosis with the classical hallmarks. Importantly, cell death was attenuated by pan-caspase inhibitors. This is consistent with earlier reports that showed that both Gamitrinib [10] and BET-inhibitors [3] on their own are capable of engaging apoptosis.

We next assessed as to how the combination treatment of Gamitrinib and BET-inhibitors activate apoptosis. To this purpose, we investigated the expression of pro- and anti-apoptotic Bcl-2 family members, showing that the combination treatment led to an increase of Noxa [18] and a decrease of Mcl-1 in most cell lines. Consequently Noxa knockdown protects from cell death induced by the combination treatment. Our proposed mechanisms of action for the combination treatment are supported by other studies, showing that a deregulated Noxa/Mcl-1 ratio is causal for cell death induction. Therapies that target Mcl-1 are considered to be valuable in view of the fact that many tumors, including glioblastomas, display high levels of Mcl-1 [19]. In turn, certain cells are Mcl-1 dependent for their survival and acute withdrawal or interference results in significant cell death. Moreover, Mcl-1 mediates resistance against certain drugs, such as Temozolomide and most evidently, BH-3 mimetics, such as ABT-737, ABT-263 [20–24] or the Bcl-2 inhibitor ABT-199 [25]. ABT-199 is the most promising out of the BH3-mimetics since it recently received accelerated FDA-approval for the treatment of chronic lymphocytic leukemia [22, 26–28].

Given that Noxa is known to be controlled by several transcription factors [29], we assessed as to whether the combination treatment of BET-inhibitors and Gamitrinib regulates Noxa at the level of transcription. Consistently, the combination treatment increased Noxa to a much higher extend than the single treatments, which was mediated by ATF4. The involvement of ATF4 in this process suggests that ATF4 appears to be a pro-apoptotic factor in this setting. While the literature suggests a dual role for ATF4 [30–34] with respect to cell death modulation, this appears to be most likely context dependent. The activation of ATF4 can be mediated by a number of factors, such as GCN2 [32] and PKR or PERK [14]. We found here that the combination treatment of BET-inhibitors and Gamitrinib mediated an increase of ATF4 via the PERK pathway, thus through endoplasmic reticulum stress. In addition, our results confirm that ATF4 is mediating the increase of CHOP [35] and Noxa [36–38] in response to the combination treatment.

The deeper questions as to how the combinatorial treatment of BET-inhibitors and Gamitrinib leads to an induction of a stress response remains to be elucidated. One possible explanation is that the combination treatment elicited metabolic stress by depleting cells of ATP. This assumption is based on the fact that c-myc and mitochondrial matrix chaperones control metabolism in tumor cells, such as glycolysis and oxidative phosphorylation [39].

Finally, we were able to demonstrate that the combination treatment of BET-inhibitors and mitochondrial matrix chaperone inhibitors is active *in vivo*. This further underscores and supports earlier findings that drug combinations, involving Gamitrinib, are efficacious and without significant toxicity. Additional clinical studies are warranted to assess as to whether this combinatorial streatment strategy is safe and efficacious in patients as well.

## MATERIALS AND METHODS

### Reagents

The BET-inhibitors, JQ1 and OTX015, were purchased from Selleckchem (Houston, TX). Gamitrinib-TPP (G-TPP) was synthesized as described in [11]. A 10 mM working solution in dimethylsulfoxide (DMSO) was prepared for all reagents prior to storage at -20°C. Final concentrations of DMSO were below 0.1% (v/v).

### Cell cultures and growth conditions

All cells were cultured as described [40–44]. GBM6, GBM14 and GBM39 human, patient-derived glioblastoma xenograft cultures originated from Dr. Jann Sarkaria (Mayo Clinic, Rochester, MI, U.S.A.). The identities of the glioblastoma cell lines we purchased were confirmed by the respective source of purchase. NCH644 glioma stem-like cells were cultured in MG-43 medium (CLS, Heidelberg, Germany) for both maintenance and experiments [40–44].

### Cell viability assays

In order to examine cellular proliferation, CellTiter-Glo^®^ assays were performed as previously described [19, 41, 45–50].

### Measurement of apoptosis and mitochondrial membrane potential

Annexin V/propidium iodide, propidium iodide and TMRE staining were performed as previously described [19, 41, 45, 47–50] or in accordance with the manufacturer instructions for TMRE staining (Cell Signaling) [10]. The data were analyzed with the FlowJo software (version 8.7.1; Tree Star, Ashland, OR).

### Western blot analysis

Specific protein expression in cell lines was determined by Western blot analysis as described before [51]. The following antibodies were used: Mcl-1 (1:500; CST: Cell Signaling Technology, Danvers, MA), human caspase-9 (1:1,000; CST), cleaved caspase 3 (1:250; CST), PARP (1:1000; CST), Bak (1:500; CST), Bcl-2 (1:500; CST), BIM (1:500; CST), ATF4 (1:500; CST), c-myc (1:500; CST), Bcl-xL (1:500; CST), Usp9X (1:1000; CST), Bim (1:500; CST), Noxa (1:500, clone 114C307; Calbiochem), β-actin (1:2,000, clone AC15; Sigma Aldrich) and secondary HRP-linked antibodies were purchased from Santa Cruz Biotechnology Inc. Some western blots were acquired, using the Azure (C300) imaging system (CCD – camera based). In some cases, capillary electrophoresis was used in lieu of western blotting. For capillary electrophoresis, the Wes instrument was used as per instructions by the manufacturer (ProteinSimple, San Jose, California, 95134 USA).

### Transfections of siRNAs

Transfections were performed as previously described [10], using either Oligofectamine or Lipofectamine 2000. PMAIP1 (encodes for Noxa) siRNA and BAK siRNA were purchased from Ambion. Two c-myc specific siRNAs were purchased from CST: Cell Signaling Technology, Danvers, MA. Non-targeting siRNA-pool (ON-TARGETplus Non-targeting Pool, # D-001810-10-05) and ATF4 (SMARTpool: ON-TARGETplus ATF4 siRNA, L-005125-00-0005) were purchased from Thermo Fisher Scientific.

### Real-time PCR and cDNA synthesis

RT-PCR and cDNA synthesis was performed as described before [51], using the following primers. 18S forward: AGT CCC TGC CCT TTG TAC ACA, 18S reverse: GAT CCG AGG GCC TCA CTA AAC, PMAIP1 forward: CTG GAA GTC GAG TGT GCT ACT C, PMAIP1 (Noxa) reverse: TGA AGG AGT CCC CTC ATG CAA G, GRP78 forward: CTG TCC AGG CTG GTG TGC TCT, GRP78 reverse: CTT GGT AGG CAC CAC TGT GTT C, C/EBPB forward: AGA AGA CCG TGG ACA AGC ACA G, C/EBPB reverse: CTC CAG GAC CTT GTG CTG CGT, XBP1 forward: CTG CCA GAG ATC GAA AGA AGG C, XBP1 reverse: CTC CTG GTT CTC AAC TAC AAG GC, CHOP forward: GGT ATG AGG ACC TGC AAG AGG T, CHOP reverse: CTT GTG ACC TCT GCT GGT TCT G.

### Subcutaneous xenograft model

1 × 10^6^ LN229 glioblastoma cells (p53 mutated) suspended 1:1 in Matrigel^®^ Matrix (Corning Inc., Corning, NY) were implanted subcutaneously into the flanks of 6–8 week-old Nu/Nu mice as as described before [41]. Tumors were measured with a caliper and sizes calculated according to the standard formula: (length * width^2^)*0.5. Treatment was performed intraperitoneally as indicated in the figure legend 6. For intraperitoneal application Gamitrinib-TPP and OTX015 were dissolved in 10% DMSO, 32% Cremophor EL (SIGMA, St. Louis, MO), 8% Ethanol (Pharmco-Aaper, Brookfield, CT) and 50% PBS.

### Statistical analysis

Statistical significance was assessed by two-tailed Student's *t*-test using Prism version 5.04 (GraphPad, La Jolla, CA). A *p* ≤ 0.05 was considered statistically significant. The CompuSyn software (ComboSyn, Inc., Paramus, NJ - www.combosyn.com last accessed 06/01/15) was used for the drug combination analysis including the calculation of the combination index (CI) [43]. A CI < 1 was considered as synergistic, a CI = 1 as additive and a CI > 1 as antagonistic.

### Study approval

All procedures were in accordance with Animal Welfare Regulations and approved by the Institutional Animal Care and Use Committee at the Columbia University Medical Center.

## SUPPLEMENTARY MATERIALS FIGURES


