# Supplementary material for: Mitochondrial matrix chaperone and c-myc inhibition causes enhanced lethality in glioblastoma
Source: Oncotarget. 2017 Mar 15;8(23):37140–53. doi: 10.18632/oncotarget.16202 (PMC5514897; doi:10.18632/oncotarget.16202)
Supplement: Supplementary file 1 [file oncotarget-08-37140-s001.pdf]

Mitochondrial matrix chaperone and c-myc inhibition causes enhanced lethality in glioblastoma

Supplementary Materials

Supplementary Table 1: G-TPP treatment elicits a synergistic anti-proliferative effect on U87 glioma cells and GBM14 patient-derived xenograft cells in the presence of the c-myc inhibitor, JQ1

U87			GBM14		
**G-TPP (µM)**	**JQ1 (µM)**	**CI**	**G-TPP (µM)**	**JQ1 (µM)**	**CI**
0.6	5.0	0.22746	0.6	5.0	0.20250
1.25	2.5	0.22814	1.25	2.5	0.41107
1.25	1.0	0.29534	1.25	1.0	0.42634
2.5	1.0	0.39435	2.5	1.0	0.67258
5.0	0.5	0.64321	2.5	2.5	0.50693
5.0	5.0	0.57001	5.0	5.0	1.15827

G-TPP treatment elicits a synergistic anti-proliferative effect on U87 glioma cells and GBM14 patient-derived xenograft cells in the presence of the c-myc inhibitor, JQ1. The CompuSyn software (ComboSyn, Inc., Paramus, NJ) was used for the drug-drug interaction analysis including the calculation of the combination index (CI). A CI < 1 was considered as synergistic, a CI = 1 as additive and a CI > 1 as antagonistic.

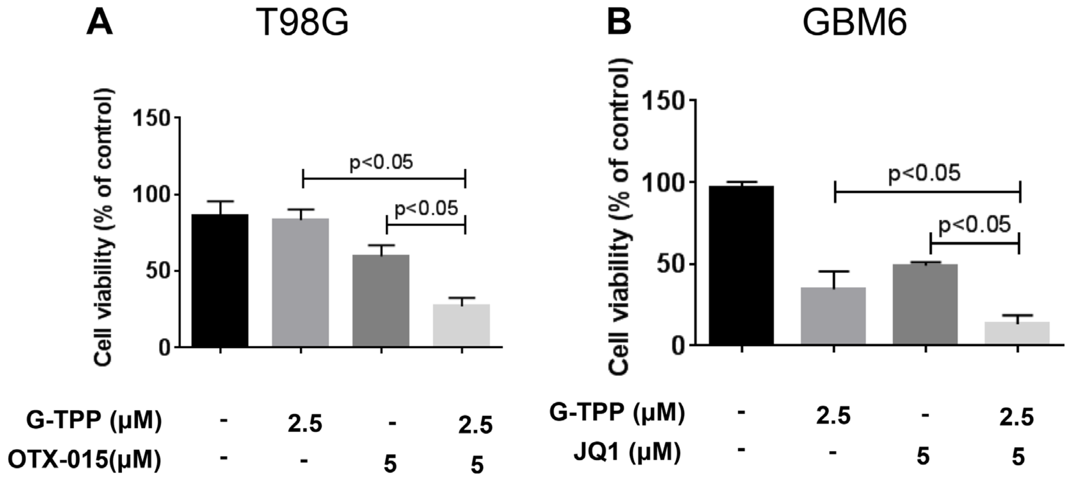

Supplementary Figure 1: The combination treatment of G-TPP and OTX015 causes synergistic reduction in cellular viability. (**A**) T98G cells were treated with G-TPP, OTX015 or the combination of both for 72 h. Cell viability was analyzed by Cell-Titer Glo assay. (**B**) Patient derived xenograft cells. GBM6, were treated with G-TPP, JQ1 or the combination of both for 72 h. Cell viability was analyzed by Cell-Titer Glo assay. *P*-values were calculated and a *p* value of less than 0.05 was considered statistically significant. Columns: mean, Error bar: standard deviation.

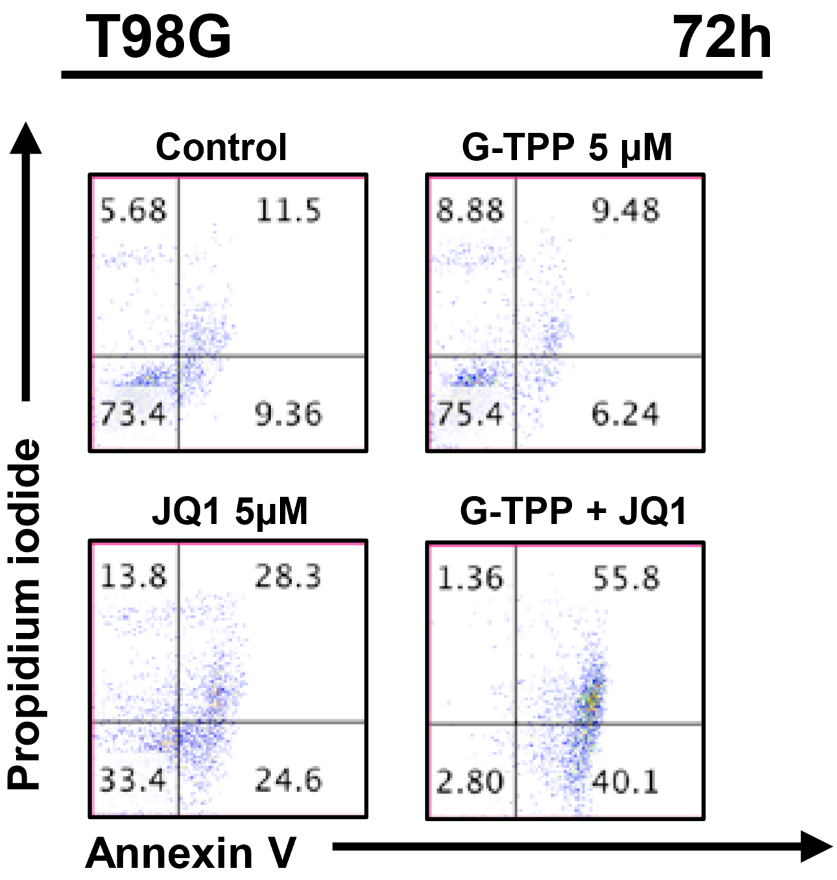

Supplementary Figure 2: The combination treatment of BET-inhibitors and G-TPP elicits enhanced apoptosis. T98G cells were treated with solvent, G-TPP, JQ1 or the combination of both. After 72 h cells were stained with Annexin V/Propidium iodide and analyzed by flow cytometry.

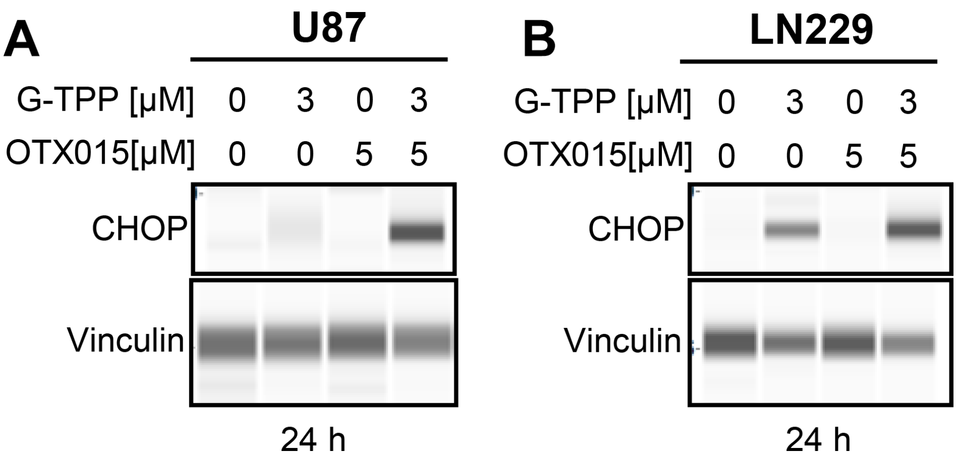

Supplementary Figure 3: The combination treatment of BET-inhibitors and G-TPP causes enhanced ER-stress. (**A**, **B**) U87 and LN229 cells were treated with G-TPP, OTX015 or the combination for 24 hours. Whole protein lysates were prepared and analzed by capillary electrophoresis on Wes (ProteinSimple) for the expression of CHOP and Vinculin.
